# Developing Methods for Maintaining Genetic Diversity in Novel Aquaculture Species: The Case of *Seriola lalandi*

**DOI:** 10.3390/ani13050913

**Published:** 2023-03-02

**Authors:** Víctor Martinez, Nicolas Galarce, Alvin Setiawan

**Affiliations:** 1 INBIOGEN, Department of Animal Production, Faculty of Veterinary Sciences, Universidad de Chile, Avda. Santa Rosa 11735, Santiago 8820808, Chile; 2 Escuela de Medicina Veterinaria, Facultad de Ciencias de la Vida, Universidad Andrés Bello, Santiago 8370146, Chile; 3 Northland Aquaculture Centre, National Institute of Water and Atmospheric Research, Ruakaka 0116, New Zealand

**Keywords:** *seriola*, inbreeding, genotyping-by-sequencing

## Abstract

**Simple Summary:**

Limiting inbreeding rates in farmed populations is crucial to ensuring long-term commercial viability. This task is particularly challenging in the aquaculture of mass communal spawning species, such as the yellowtail kingfish (*Seriola lalandi*). This reproductive strategy often results in a skewed parental genetic contribution while introducing additional complexities in parentage determination (c.f., controlled matings). To overcome these issues, we developed a marker panel based on genotyping-by-sequencing spanning 300 SNPs for parentage determination. Panel performance was satisfactory, which advocates for its employment to increase the long-term sustainability of this aquaculture resource when implementing breeding programs.

**Abstract:**

Developing sound breeding programs for aquaculture species may be challenging when matings cannot be controlled due to communal spawning. We developed a genotyping-by-sequencing marker panel of 300 SNPs for parentage testing and sex determination by using data from an in-house reference genome as well as a 90 K SNP genotyping array based on different populations of yellowtail kingfish (*Seriola lalandi*). The minimum and maximum distance between adjacent marker pairs were 0.7 Mb and 13 Mb, respectively, with an average marker spacing of 2 Mb. Weak evidence of the linkage disequilibrium between adjacent marker pairs was found. The results showed high panel performance for parental assignment, with probability exclusion values equaling 1. The rate of false positives when using cross-population data was null. A skewed distribution of genetic contributions by dominant females was observed, thus increasing the risk of higher rates of inbreeding in subsequent captive generations when no parentage data are used. All these results are discussed in the context of breeding program design, using this marker panel to increase the sustainability of this aquaculture resource.

## 1. Introduction

The yellowtail kingfish (*Seriola lalandi*) is a pelagic carnivorous fish that inhabits tropical and temperate waters of the Southern Hemisphere and the Northern Pacific, with known populations in Australia, New Zealand, Japan [1], the Southeast China Sea [2], the Mediterranean Sea [3], and the Pacific coast of South America [4,5,6]. As the demand for yellowtail kingfish continuously grows and fishery quotas have reached maximum levels [3], commercial aquaculture production of this species has been successfully established in Australia [7], the Netherlands, and Denmark, while the establishment of new farms has been planned in New Zealand [8] as well as North and South America [9,10].

The complete production cycle of *S. lalandi* has been successfully set up along the northern coast of Chile [10]. However, relatively little attention has been paid to a proper understanding of the genetic structure and diversity of both natural and captive populations of this species. In countries such as Germany, the Netherlands, and the USA, commercial aquaculture has been established using juveniles from single farms (particularly from Chile, Juan Lacamara, Pers. Comm.), where broodstock were initially sourced from natural fisheries, often with little or no attention to genetic diversity [4]. Nevertheless, such practices are not compatible with the long-term viability of the industry [11].

Traditional pedigree-based breeding programs for *S. lalandi* can also prove challenging. Similar to many other species, *S. lalandi* is a communal broadcast spawner that readily breeds in captivity without requiring hormonal inductions or gamete stripping for in-vitro fertilization (IVF) [7]. While this trait is highly beneficial for practical husbandry and production purposes, the absence of controlled matings prevents the holding of progeny by family, which allows for simple (non-molecular method) pedigree tracking. Combined with their high fecundity rate and propensity for skewed parental contribution [7], there is a high risk that high inbreeding rates arise in breeding programs, leading to a rapid reduction in effective population size (Ne) [12,13,14,15,16,17]. Furthermore, the difficulty in securing an even representation of all the broodstock in single production batches adds complexity for accurately assessing genetic parameters. For example, heritability and genetic correlations for harvest traits estimated in a commercial population were in most cases not statistically significant, which is likely due to the low number of parents (8 sires and 6 dams) used for estimation of such parameters [18,19].

For this reason, it is necessary to develop accurate marker panels to assess parentage while using pedigrees as a tool to control the rates of inbreeding and maintain genetic diversity in sustainable breeding programs. Genetic studies for paternity testing have so far been carried out using microsatellite markers developed from other species of the same genus [4,20]. Using heterologous microsatellites can introduce biases when assessing population variability; for instance, null alleles and homoplasy may falsify the genetic structure and parentage testing. 

The aim of this study is to develop a novel genotyping-by-sequencing (GBS) marker panel for parentage testing using single nucleotide polymorphism (SNP) data obtained from a genotyping array and a whole *S. lalandi* genome assembly, as presented elsewhere [21]. We focused our attention on the performance of this panel under real experimental conditions, considering the information of progeny data from two source populations, one native to Chile and another to New Zealand (NZ). This information was used to calculate predicted inbreeding rates using the genetic contribution theory and variation in family size. The results are discussed from the perspective of developing breeding programs for “these new” aquaculture species that are effective in increasing the genetic gain while constraining the rates of inbreeding.

## 2. Materials and Methods

### 2.1. Production System and Data Sampling

Fish from Chile were sampled from a captive *S. lalandi* broodstock population held in Acuinor S.A. Company’s facilities in Caldera City, located in the Atacama Region. These fish were captured from a wild population at Punta Frodden as well as from different locations near Caldera (27.0° S; 70.8° W) and kept to conform to the base population of the national *S. lalandi* breeding programs (56 dams and 51 sires). The individuals were arranged in four independent breeding units (R1, R2, R3, and R4 with about 20–30 fish per tank). Hence, they were exposed to different photoperiods and temperature increases to ensure the availability of larvae throughout the entire year. The progeny was reared under standard farm conditions, and the fish were kept until harvest at about 3 Kg in different Recirculating Aquaculture Systems (RAS) units. We sampled fin clips from all Chilean broodstock and 161 randomly sampled progeny from the different breeding units (R1, R2, and R3) produced by mass spawnings. All fish were anaesthetized before sampling using MS-222 as part of the management plan to measure production variables.

Fish from New Zealand (*n* = 31) were sampled from a captive broodstock originated from the east coast of the Northland province (35° S, 174° E) North Island and was kept in captivity for 2 to 8 years. The animals had been held at the NIWA Northland Aquaculture Centre, located in Ruakaka. 

Fin clips were kept frozen or in >70% ethanol before DNA extraction. Genomic DNA was extracted using the NucleoSpin^®^ Plant II kit (Macherey-Nagel^®^, Düren, Germany) according to the manufacturer’s instructions. The samples were quantified using a Qubit fluorometer (Thermo Fisher Scientific, Waltham, MA, USA) with the Qubit dsDNA BR Assay Kit (Thermo Fisher Scientific, Waltham, MA, USA). DNA was normalized to a final concentration of 2 ng/µL.

### 2.2. Genotyping-By-Sequencing (GBS) Panel Construction

The marker panel was developed using the genomic resources within the national breeding program of *S. lalandi*. To develop the genetic resources needed for its implementation, we first produced an in-house genome assembly for the species. This reference was developed by sequencing a single male with a coverage of 70× using a mixture of single and mate Illumina pair-ends reads. The draft genome was assembled using MaSuRCA [22] with the default parameters. An additional 34 individuals (17 males and 17 females) were sequenced at 10× coverage to discover SNPs using the scaffolds obtained by MASURCA and the procedures explained below. These fish were also used to discover SNPs associated with the sex determination gene, that appeared to be causal [23]. We used 90 K SNPS selected (out of more than 5 million SNPs discovered) to develop the 90 K-SNP genotyping array (constructed by Thermofisher^TM^, Waltham, MA, USA). Scaffolds were anchored to linkage groups with CHROMONOMER [24] using the linkage distance between 90 K SNPs markers as obtained from the genotyping array using LEP-MAP (we used information on 200 progeny from 10 full sibs families to generate the linkage map [25]). The assembled genome comprised 24 linkage groups with a total genome size of about 657 Mb encompassing 95% of the total expected genome. More detailed information will be provided in a separate study (in preparation [23,26]). 

**Figure 1:**
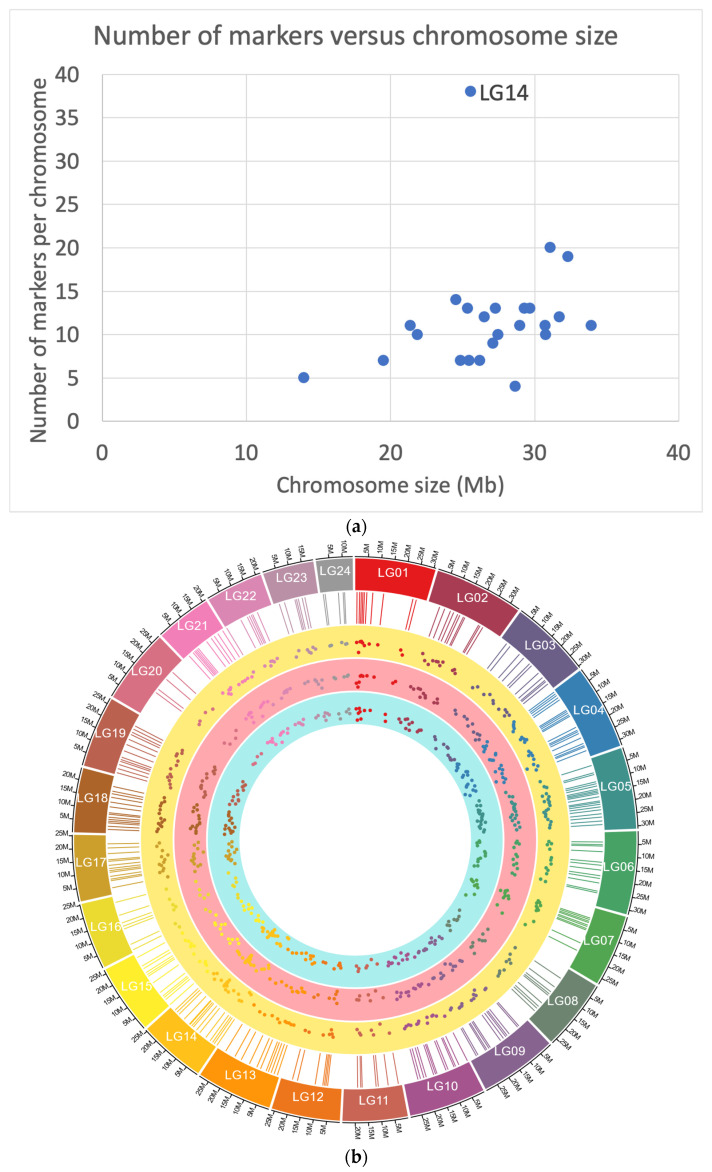
(**a**) Number of markers per chromosome (Y-axis) versus chromosome (linkage group: LG) size in Mb (assume 1cM equals Mb: X-axis). The linkage group 14 (LG14) included the markers for sex determination. (**b**) Markers used for the GBS panel and MAF. The outer layer shows the genome, the second layer (rectangle) shows the position of the markers, while the third, fourth, and fifth layers the observed MAF for the New Zealand and Chilean populations.

Selected markers used for the construction of the GBS panel were obtained from genotype data of the base (founder) population of the national Chilean breeding programme of *S. lalandi*. In this case, a total of 300 SNPs were selected using genotype data obtained from the 90 K-SNP-genotyping array of the *S. lalandi* broodstock population [23] as obtained from data from the broodstock population genotyped with the 90 K-SNP-genotyping array. The SNPs (280 markers) used for parentage assignment in the panel were evenly placed across all 24 linkage groups. The markers were selected based on informativeness using a minimum allele frequency (MAF) of 0.34. The average marker spacing is 2 Mb (the minimum and maximum distances between pairs of markers were 0.7 Mb and 13 Mb, respectively; see Figure 1). All the markers selected for the panel followed Hardy-Weinberg equilibrium in the broodstock population and showed no linkage disequilibrium within linkage groups. We included 20 markers in the vicinity of the diagnostic SNP used for sex prediction (these markers were obtained from a genome-wide association analysis as explained above and will be published in a separate study [23,26]).

For obtaining the actual genotypes of the progeny, a targeted GBS protocol was used. A total of 300 primer pairs were developed based on proprietary software from Thermo Fisher (https://www.thermofisher.com/cl/es/home/global/forms/agriseq-breeding.html (accessed on 20 January 2021); Thermo Fisher Scientific, Waltham, MA, USA). A total of 192 libraries were prepared using AgriSeq™ HTS Library Kit (Thermo Fisher Scientific). Libraries were genotyped using the Ion 540 Chef kit along with the Ion 540 Chip (~80 million reads) kit (Thermo Fisher Scientific). Sequences were mapped to the *S. lalandi* genome assembly using BWA [27]. SAM files were sorted with SAMtools [28], and PCR duplicates were removed using SAMBAMBA [29]. SNPs were identified using FreeBayes (https://arxiv.org/abs/1207.3907v2 accessed on 30 March 2021) with default settings. The initial set of SNPs identified was filtered using vcftools [30] based on the following criteria (derived from the FreeBayes output): (1) a Phred-scaled SNP quality score with significance greater than 30; (2) minimum allele frequency of 0.49; (3) maximum percentage of missing values of 0.20; and (4) the maximum number of alleles as 2.

### 2.3. Genotype Detection and Parentage Analysis Using the GBS Panel

SNP variation statistics were obtained separately for the different datasets (New Zealand (*n* = 31) and Chile (*n* = 161)) when using the GBS panel. We calculated minimum allele frequency (MAF), Hardy-Weinberg χ^2^ statistics, and observed heterozygosity (He) using PLINK [31]. The cumulative probability of exclusion (CPE) for single-parent (CPE-1) and both-parent inference (CPE-2) were calculated using formulae of Jamieson and Taylor [32], as well as the polymorphic information content (PIC) with equations obtained from [33].

Parentage assignment was carried out using data from the Chilean broodstock (which was genotyped by the 90 K genotyping array) and progeny (161, as explained above) with AlphaAssign [34]. This procedure relies on a maximum likelihood approach to infer parents (sires or dams) using default parameters. In practice, sex is predicted at the progeny level with the GBS marker panel, making it possible to independently assign sires or dams in the next generation when selecting potential broodstock from the progeny available. This is expected to increase the accuracy of the procedure by reducing the number of possible single-parent (sire or dam) and progeny pairs when calculating the likelihood [35]. Since we did not use the GBS panel for genotyping the parents, we obtained genotypes of the parental generation by extracting the markers selected for the GBS panel from data obtained from genotypes of broodstock using the 90 K-SNP-genotyping array.

The predicted rates of inbreeding were calculated using two different methods. The first one uses genetic contribution theory, assuming random selection and discrete generations [36]. In this case, an estimate can be obtained by using the following approximation (Equation (1)):

(1) ΔF=14[∑ ri2]

where $r_{i}$ is the mean parental genetic contribution for each parent calculated, from generation 0 to generation 1, using the predicted pedigree. The mean genetic contributions were obtained from the additive relationship values between parents and progeny and then averaged over the total number of progenies. The values of $r_{i}$ summed over each sex (dam or sires) equal 0.5.

We also used the method derived by [37] for estimating inbreeding effective size that incorporates the offspring contribution (Equation (6), without selfing from [37]), as:

(2) $$Ne=\frac{2S-2}{\frac{\sum^{\;}(k_{i}^{2})}{2S}-1}$$

where *S* is the total number of progenies, and *k_i_* is the family size for each of the parents (males and females). This method is subject to relatively large standard errors when the number of parents is large and the number of offspring is small (giving an upward bias to *Ne*, as obtained from simulations [37]). The estimates of the rates of inbreeding and effective size were obtained as follows:

(3) ΔF=12Ne

The effective numbers of founders (*ENF*), was calculated as [38]:

(4) $$ENF=1/\left[\sum^{\;}r_{i}\right]\;$$

All these calculations were carried out using PEDIG [38] and Excel spreadsheets, using the pedigree predicted with AlphaAssign. Estimates of *Ne* and predicted rates of inbreeding were obtained using information from all the breeding units together as well as separately.

## 3. Results

### 3.1. Genotyping-By-Sequencing (GBS) Marker Panel Assessment

We assessed the informativeness of the GBS marker panel across the different populations analyzed (New Zealand and Chile). After sequencing, a total of 188 out of 192 samples passed with a minimum sample call rate of 97% (with an average of 98%). The average coverage was 1152× per marker. Seven SNP markers did not pass the quality control since they had a low call rate and were excluded. One additional marker showed a MAF equal to 0 in the progeny. Therefore, a total of 272 markers were mapped to the 24 chromosomes using BWA (Supplementary Table S1; Figure 1a). These markers were used in the final parentage analysis based on the GBS panel, and 20 markers were used for sex prediction. The list of SNPs and their detailed information is given in Supplementary Table S1.

When examining the 272 markers used for paternity testing, MAF values ranged from 0.11 to 0.50, with an average of 0.36 for the entire dataset (Figure 1b). The percentage of SNPs with a MAF between 0.40 and 0.50 was 36% and 40%, respectively, for the Chilean and New Zealand populations. In addition, the average polymorphic information content (PIC) was 0.50 and 0.53 for the Chilean and New Zealand populations, respectively. The realized average linkage disequilibrium (r^2^) between adjacent marker pairs within chromosomes was 0.05 and 0.04 for the Chilean and New Zealand populations, respectively. In addition, the CPE for single-parent (CPE-1) and both-parent (CPE-2) inference were in all the populations higher than 0.999. 

### 3.2. Parentage Testing and Distribution of Genetic Contributions

**Figure 2:**
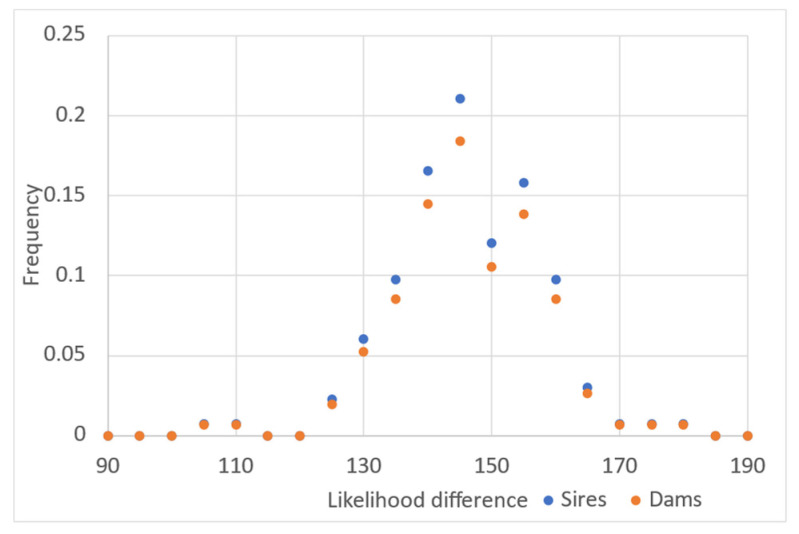
Histogram of the likelihood difference between the chosen candidate (sires or dams) and unrelated sires or dams for each progeny evaluated using all markers included in the panel (272 SNPs).

The average likelihood difference between unrelated parents (sires or dams), which is calculated to assign parentage, was substantially higher than in the case of chosen candidate parents using all marker data (these values were calculated with AlphaAssign using default parameters, Figure 2). The parents that were not assigned to offspring had values for the likelihood difference near zero. We tested individuals from New Zealand (as progeny) with putative parents from Chile. In this analysis, no putative sires or dams were assigned to the individuals coming from the New Zealand sample population, so the rate of false positives was negligible (data not shown). 

**Figure 3:**
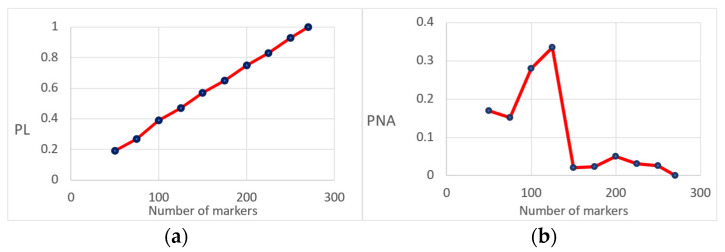
(**a**) Ratio of the average difference between the likelihood (PL) of the chosen parent and an unrelated individual for a different number of markers (expressed as a proportion when using all markers); (**b**) Proportion of non-assignment (PNA) given a different number of markers used for parentage testing.

We tested different scenarios to assess the effect of the number of markers and the performance of the maximum likelihood approach for assessing parentage (Figure 3). This was achieved by randomly deleting markers used to infer parentage with PLINK using the thin option. We found a linear trend between the number of markers and the difference between the likelihood for a specific set of markers and the full set of markers (the ratio of the average difference between the likelihood for a reduced set of markers and the full set of markers was 20 to 100%). The linear regression coefficient was about 0.04 units per marker (Figure 3a). The probability of non-assignment (using the number of parents not assigned due to a reduced likelihood difference from the total number of parents assigned) is highly dependent on the number of markers. The threshold for accurately assigning parental data was about 150 markers. (See Figure 3b, in which the value of the proportion of non-assignment remains at a maximum value of 5% after 150 markers).

**Figure 4:**
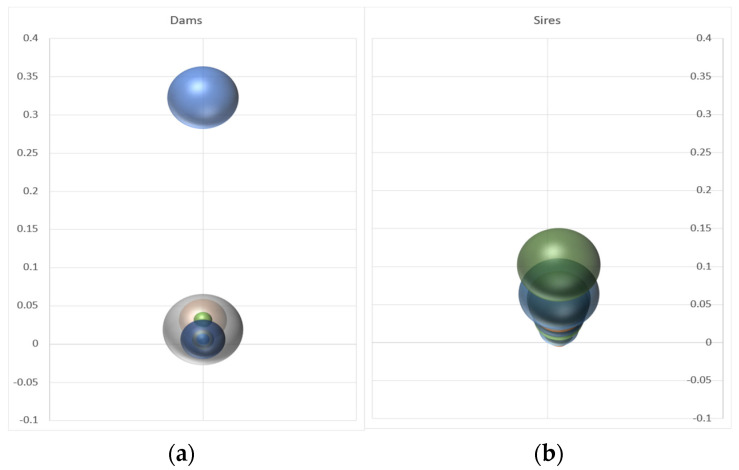
Bubble graphs showing the mean genetic contributions of single dams (**a**) and sires (**b**) across different breeding units. The Y-axis denotes the proportion of offspring per dam and sire.

The parental assignment revealed an extreme asymmetry in the parental contributions, particularly concerning the females (Figure 4). In the more extreme case, a dam was indicated as the single parent of almost all the progeny in one of the production batches (see Figure 4 and Table 1). The mean number of offspring per dam (17 out of 55 dams were assigned) was 12, with a very high variance (293). The males contributed more evenly to the gene pool. The average offspring count of males (36 out of 51 males were assigned) was 6, but with a much lower variance (24). 

The predicted rates of inbreeding varied significantly between methods, especially when considering individual breeding units. Overall, it was lower when using the method using genetic contributions (Table 1). Nonetheless, the predicted rates of inbreeding were in general very high when considering specific production groups, which is not surprising given the very low numbers of parents contributing within each batch, ranging between ∆F_1_ 4% and 7% and ∆F_2_ ranging between 6% and 12% (Table 1). When considering all the groups together, the predicted rate of inbreeding was about 2.1% and 3.3% for DF1 and DF2, respectively. This gave the values of the effective population sizes of about 23.8 and 15.1 for *Ne*_1_ and *Ne*_2_ (see Table 1). The effective number of founders was about one-third the number of breeders contributing progeny in the next generation (Table 1). 

## 4. Discussion

Molecular parentage testing in aquaculture has been traditionally carried out using microsatellites [20]. However, the general lack of species-specific standardized panels prevents efforts to automate the process, resulting in microsatellites not being routinely used for parentage analyses as a tool to control inbreeding. Furthermore, many microsatellites developed on congeners (e.g., *S. quinqueradiata*) are not usable due to their low informativeness and the presence of null alleles. Indeed, our research team showed that 10 out of 25 non-focal microsatellites are useless for parentage analysis [20]. Additionally, microsatellite panels are not useful for sex determination, requiring a separate step of high-resolution melt PCR [21]. For all these reasons, it is important to develop marker panels using SNPs, which are faster to obtain, more accurate, and cheaper when compared with microsatellites.

In this study, the realized value for the cumulative probability of exclusion for a single or both parents was 1, suggesting that the GBS marker panel of 272 markers can be used very efficiently for parentage testing in captive *S. lalandi* populations. In fact, when using only 150 SNPs as markers, there is enough power to perform parentage analysis (Figure 3), with a rate of non-assignment lower than 5%. We also found that false positives are rare, since when performing parentage testing on an unrelated population, no related parents were obtained when more than 150 markers were used.

### 4.1. Predicted Rates of Inbreeding in S. lalandi

The predicted rates of inbreeding calculated in this study should be interpreted as long-term estimates and not as forecasts of average inbreeding in the next generation. Furthermore, these rates should be thought of as rough estimates as they only rely on information from a relatively low number of single spawning events over limited time spans. Therefore, the genetic contributions are not at their asymptotic values, leading to an underestimation of the rates of inbreeding in the long term. This can be seen in Table 1, where values based on genetic contributions are smaller than estimates from another study [37]. 

Some unintentional selection is likely ongoing in the population since, in large production batches, individuals are graded to optimize feeding practices while decreasing the rates of cannibalism [39]. This preselection phase may explain, to a certain degree, the high variance of some of the parents assigned. Nevertheless, specific females seem to dominate single spawning events since they appear in half-sib maternal families with several males, which is in accordance with what had previously been observed in this species [40]. Therefore, inbreeding is of concern in this species since, without intervention, it will be extremely high within a short time, and strategies to control its rates are needed.

Overall, the predicted inbreeding rates in our breeding units were generally very high. In all cases, values of the effective population size were much smaller than the ones required to reduce the risk of extinction in species with high reproductive output [14,35,41]. In practice, a cumulative number of spawnings should be used in order to secure a sustainable breeding program (see below). This means that the generation interval will be higher when compared with other species (i.e., salmonids), since multiple egg batches should be kept over longer time spans.

There is some scope to constrain the rates of inbreeding by avoiding mating between related individuals, at least in the short term. In our case, since the founders of the population are the actual broodstock used for parentage testing, it is straightforward to select replacements based on the minimum inbreeding (using the progeny candidates) using a rotational mating scheme. This system has been devised in the classical paper by Kimura & Crow [42] and applied to Coho salmon (*Oncorhynchus kisutch*) to replace broodstock [43]. Nevertheless, this methodology would only be useful for the short term, and a high rate of inbreeding has been observed in the long term [14]. In this mating system, broodstock was kept in a few separate groups, and males were transferred sequentially and circularly between neighboring groups, which were kept isolated [44]. In the simplest system to be considered, *n* males and *n* females were arranged alternately so that each potential sire or dam mated with individuals of a neighboring reproduction unit. In this classical system, the second generation is the product of a half-sib or full-sib mating (when dams are also replaced). We have applied this replacement method by sequentially assigning sires and dams to unrelated reproduction units (R1, R2, R3, and R4). This procedure gave null inbreeding in the second generation, as expected since the co-ancestry between breeders within tanks was still 0 (only unrelated founders are available in generation 0). Further investigation is needed to understand the consequences of this type of mating in the long term. 

### 4.2. Low-Density Marker Panels, Genomic Prediction, and Two-Stage Selection Programmes

We have shown that specific progeny batches represent a reduced number of founders from the previous generation, even though the number of progenies can be large (since potentially millions of eggs are produced in each spawning event). This situation will lead to a series of practical problems when implementing genomic breeding programs in practice. 

First, if a reduced number of batches are produced in each generation, it will be difficult to predict accurate breeding values using SNPs since the associations between markers and quantitative trait loci (QTL) would not represent associations at the population level (most of the information will come from a small number of full-sib families). Secondly, progeny from single spawning events are related to half-sib maternal families, and therefore (when mated), they will produce inbred offspring. In the case of *S. lalandi*, several batches are produced within a year, but the progeny of each batch [30] mostly originates from a single dominant female (Figure 4 and [20,40]). For these reasons, it is important to include selection candidates from as large a number of batches as practicable in order to represent a significant proportion of the genomic variation at the population level. 

Secondly, one major issue in developing breeding programs for *S. lalandi* using genomic information is the genotyping cost of using, in particular, SNP-genotyping arrays. The price of the GBS marker panel is about 10% of the cost of the full genotyping array, giving some scope for implementing profitable selection programs, as we detailed as follows. 

We have previously found that in salmonids, a two-stage selection program gave the most profitable breeding results at the expense of maximum genetic gains and constrained rates of inbreeding [45]. Therefore, an essential part of the design should follow a procedure for maintaining high levels of genetic variability by keeping a larger number of production batches in the first stage of selection while selecting for the traits of interest in different stages of the life cycle. This can be completed in the early stages, when the fish are still comparatively small in size and, hence, easier to manage (when fish can be handled in large batches from different spawnings at lower body weights, for example, at 10 months). At this stage, a set of diverse production batches can be sampled based on the trait of interest and then subjected to paternity testing for culling related individuals, based on a procedure minimizing coancestry levels between chosen candidates. The parentage testing using the GBS marker panel in this stage is essential to reconstruct the pedigree of the population and secure enough individuals of each sex differing by some degree of sexual dimorphism for body weight (females appear to be heavier than males during maturation) [46].

A second stage should be performed by selecting individuals based on the genomic breeding values of the selected population while applying optimal contribution selection [47]. In this case, breeding values should be predicted for harvest weight (usually 3–5 kg.) using information from a reference population and a denser genotyping array or a modification of a GBS genotyping incorporating SNP associated with traits of interest or using imputation. The exact proportions of individuals to be used for either stage require further investigation, as does the assessment of the optimum selection intensity to maximize profit [45,48].

Another possibility is to use a smaller number of markers in genotyping panels for selecting individuals in a single stage (usually 3–5 kg) using imputation. This will decrease the genotyping costs while possibly maintaining the rates of gain, as has been suggested before [49]. Nonetheless, it is difficult to constrain the inbreeding rate in these scenarios while using a reduced number of production batches for selecting individuals as potential selected broodstock candidates. Further research on this subject is required to disentangle the factors enabling sustainable rates of genetic gain. 

## 5. Conclusions

In this study, we developed a marker panel to perform parentage testing as well as sex determination (“Martínez manuscript in prep.”) in *S. lalandi*. This resource is essential when implementing breeding programs, since controlling inbreeding rates requires not only parentage testing but also predicting candidate sex. We used markers spanning all the linkage groups devised using recombination data from a reference genome. This enabled us to select markers with high variability and minimal linkage disequilibrium. The validation in two unrelated populations (New Zealand and Chile) suggests that only half of the markers are required for parentage testing with sufficient power. No false positives were detected when considering cross-parentage testing between populations. This panel is key to developing sound breeding strategies that constrain the rates of inbreeding in the short and long term. This is particularly important considering the difficulties in carrying out breeding programs in species subjected to communal rearing and uncontrolled reproduction. These achievements will increase the viability of this new aquaculture resource and, as such, the success of the associated business activities.

## Figures and Tables

**Table 1:** Predicted rates of inbreeding and effective population size (*Ne*) for different production batches. DF_1_ (Predicted rate of inbreeding: Wooliams and Thompson, 1991); *Ne*_1_ (*Ne*: Wooliams and Thompson, 1991); DF_2_, (Predicted rate of inbreeding: Waples and Waples, 2005); *Ne*_2_ (Waples and Waples, 2005); ONF (Observed number of females); ON_M_ (Observed number of males); N_F_ (Number of contributing females); N_M_ (Number of contributing males); ENF, (Effective number of founders); E_kf_ (Average family size of females); E_km_ (Average family size of males); V_kf_ (Variance of family size of females); V_km_ (Variance of family size of males). R3′ corresponds to a different production batch from the breeding unit R3.

Breeding Unit	ΔF1	*Ne*_1_	ΔF2	*Ne*_2_	ONF	ONM	NCF	NCM	ENF	E_kf_	E_km_	V_kf_	V_km_
R1	0.06	7.9	0.12	4.1	18	13	5	9	4	7	4	192	17
R2	0.06	8.6	0.09	5.8	15	9	5	10	4.9	12	3	292	6
R3	0.07	7.4	0.11	4.5	13	15	3	6	3.7	12	4	96	1
R3′	0.04	12.7	0.06	9.2	13	15	7	14	7.1	9	3	115	21
Overall	0.02	23.8	0.03	15.1	46	35	17	41	13.3	12	6	293	25

## Data Availability

The marker data is available upon request to the corresponding author. All the primers used for sequencing will be available for sequencing using Ion 540 chef upon request. The reference genome will be available upon request.

## Supplementary Materials

The following supporting information can be downloaded at: https://www.mdpi.com/article/10.3390/ani13050913/s1, Table S1: Position and Minimum allele frequency for each SNP used in paternity testing. The position was obtained by using [26].
